# Managing spatio-temporal heterogeneity of susceptibles by embedding it into an homogeneous model: A mechanistic and deep learning study

**DOI:** 10.1371/journal.pcbi.1012497

**Published:** 2024-09-30

**Authors:** Biao Tang, Kexin Ma, Yan Liu, Xia Wang, Sanyi Tang, Yanni Xiao, Robert A. Cheke

**Affiliations:** 1 School of Mathematics and Statistics, Xi’an Jiaotong University, Xi’an, People’s Republic of China; 2 The Interdisciplinary Research Center for Mathematics and Life Sciences, Xi’an Jiaotong University, Xi’an, People’s Republic of China; 3 School of Mathematics and Statistics, Shaanxi Normal University, Xi’an, People’s Republic of China; 4 Natural Resources Institute, University of Greenwich at Medway, Central Avenue, Chatham Maritime, Kent, United Kingdom; 5 Department of Infectious Disease Epidemiology, Imperial College London, School of Public Health, White City Campus, London, United Kingdom; Animal and Plant Health Inspection Service, UNITED STATES OF AMERICA

## Abstract

Accurate prediction of epidemics is pivotal for making well-informed decisions for the control of infectious diseases, but addressing heterogeneity in the system poses a challenge. In this study, we propose a novel modelling framework integrating the spatio-temporal heterogeneity of susceptible individuals into homogeneous models, by introducing a continuous recruitment process for the susceptibles. A neural network approximates the recruitment rate to develop a Universal Differential Equations (UDE) model. Simultaneously, we pre-set a specific form for the recruitment rate and develop a mechanistic model. Data from a COVID Omicron variant outbreak in Shanghai are used to train the UDE model using deep learning methods and to calibrate the mechanistic model using MCMC methods. Subsequently, we project the attack rate and peak of new infections for the first Omicron wave in China after the adjustment of the dynamic zero-COVID policy. Our projections indicate an attack rate and a peak of new infections of 80.06% and 3.17% of the population, respectively, compared with the homogeneous model’s projections of 99.97% and 32.78%, thus providing an 18.6% improvement in the prediction accuracy based on the actual data. Our simulations demonstrate that heterogeneity in the susceptibles decreases herd immunity for ~37.36% of the population and prolongs the outbreak period from ~30 days to ~70 days, also aligning with the real case. We consider that this study lays the groundwork for the development of a new class of models and new insights for modelling heterogeneity.

## Author summary

This study developed a novel modelling framework that explicitly integrates spatial heterogeneity into traditionally homogeneous compartmental models for precise and efficient prediction of epidemics of infectious diseases. We employed the deep learning method to learn the recruitment rate of susceptible individuals from data, given its inherent complexity and limited information. Moreover, we predefined the mechanistic form of the recruitment rate to facilitate interpretation and scenario analysis, where we fitted the recruitment rate of the mechanistic model to those obtained from the deep learning-based approach. Compared to the homogeneous model, our mechanistic model predicts significantly lower peak and final sizes of the epidemic, as well as reduced levels of herd immunity. Using real data as a benchmark, our model’s predictive accuracy has markedly improved. The framework of our mechanistic model employs a straightforward approach to integrate heterogeneity into compartment models, thereby facilitating the extension of the modelling framework to encompass various aspects of infectious disease transmission with spatio-temporal heterogeneity among the susceptible population.

## Introduction

Over the past few decades, emerging infectious diseases, such as SARS, influenza A H1N1, and SARS-CoV-2, have posed significant threats to global public health [1–3], and the control of such infectious diseases has garnered considerable attention from researchers [4,5]. Mathematical models play a crucial role as tools for understanding the transmission mechanisms of emerging infectious diseases, predicting epidemic trends, and evaluating transmission risks. These models can aid in optimizing control strategies to enhance global public health and reduce economic costs [6–8]. In particular, the precise prediction of epidemic trends serves as a foundational basis for decision-making in the implementation of control interventions. However, there are numerous challenges associated with achieving accurate predictions, with heterogeneity being one of the significant obstacles to achieving precise predictions.

**Fig 1:**
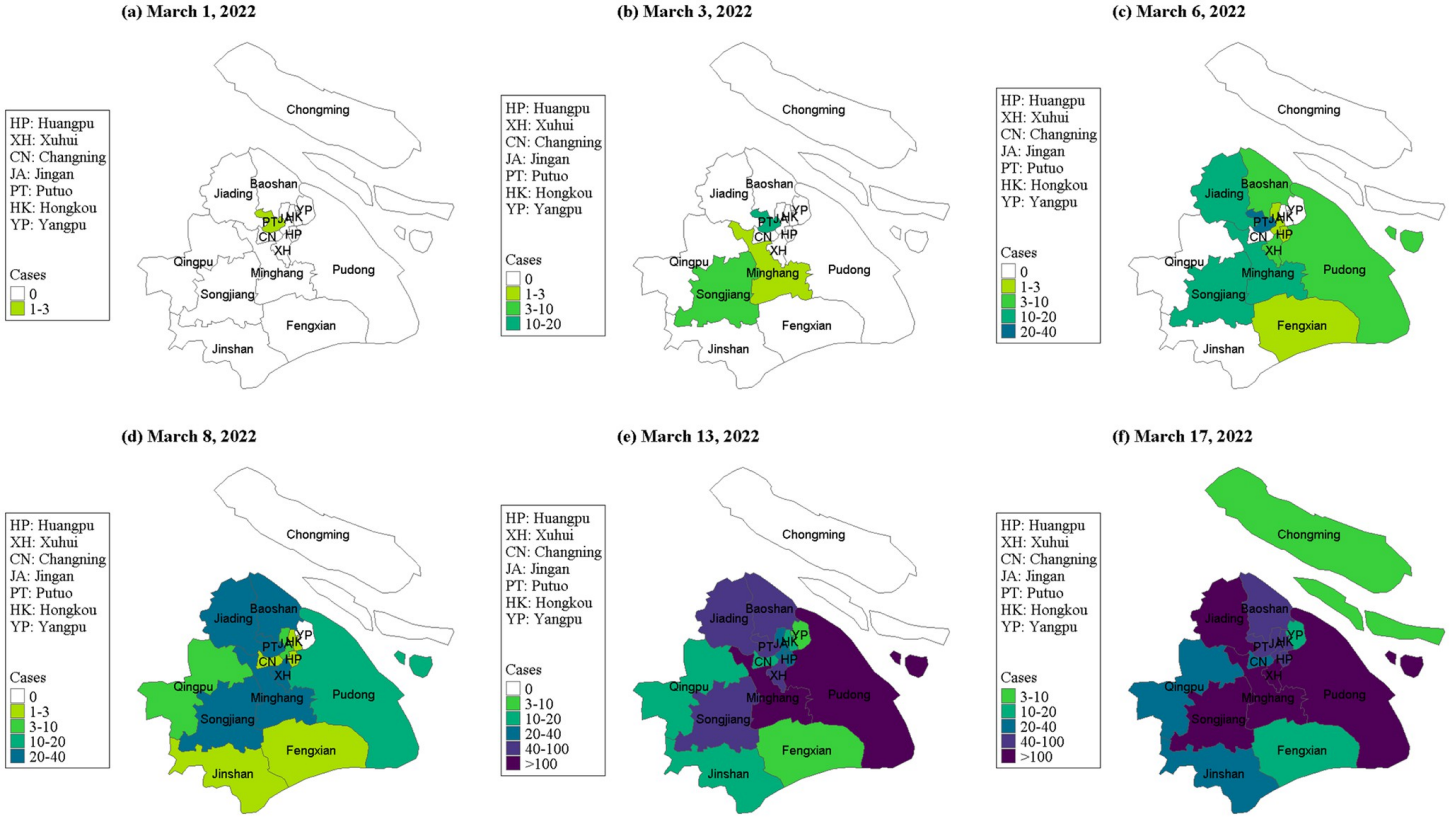
Heat-map of the cumulative numbers of infected cases showing the spatial spread of the Omicron variant of COVID-19 during the initial phase of the outbreak in Shanghai city during 1–17 March 2022. The source of the basemap shapefile was from the open access platform: National Platform for Common Geospatial Information Services (https://www.tianditu.gov.cn/; note that this link works well in P.R. China, but may be blocked in some other countries).

A susceptible population is usually defined as comprising those individuals who can be infected by a virus or other pathogen. The transmission of pathogens occurs through several mechanisms: one is direct transmission involving physical contact between infectious individuals and the susceptible population, as seen with many respiratory viruses. In another mechanism, unique to vector-borne diseases transmission occurs when an infected vector, such as a mosquito, bites a susceptible human. Sexually transmitted diseases are spread through sexual contact, which can include any form of sexual activity involving the exchange of body fluids. However, regardless of the transmission mechanism, the simplest SIR model structure assumes that all susceptibles are equally likely to be infected by infectious individuals, even though many of them may not have the opportunity to come into contact with an infectious individual simultaneously due to a heterogeneous contact structure or spatial processes. Therefore, when considering the spatio-temporal heterogeneity of susceptibles, the actual susceptible population in homogeneous models should consist of individuals actively involved in the transmission process—those with an approximately equal probability of encountering infectious individuals. Moreover, additional susceptible individuals may have increased contact with infectious individuals as the epidemic spreads spatially. This phenomenon is intuitively observed in nearly all outbreaks of SARS-CoV-2 (henceforth COVID-19) and other infectious diseases. For instance, Fig 1 illustrates the spatio-temporal shift of confirmed cases during a local outbreak of the Omicron variant of COVID-19 in Shanghai, China, from 1 to 17 March 2022. In this scenario, the Chongming district of Shanghai reported its first case on 17 March 2022–17 days after the diagnosis of the initial confirmed case in the Putuo district on 1 March 2022. This observation underscores the dynamic nature of the susceptible population and its involvement in the transmission process as infections spread over space and time. The significance of spatial heterogeneity has long been acknowledged in epidemic modelling, highlighting the limitations of homogeneous assumptions [9,10]. Consequently, numerous studies have explored various methods to incorporate spatial heterogeneity, yielding a wealth of intriguing findings [11–14].

Patch, network and spatial reaction-diffusion models can serve as frameworks for modelling the intricacies of spatial heterogeneity. Numerous existing studies have demonstrated that these modelling frameworks qualitatively enhance our understanding of the underlying transmission mechanisms of infectious diseases [11,12,15,16]. Nevertheless, there are challenges in establishing connections between these models and real-world data, conducting quantitative analyses, and making predictions due to their inherent structural complexity. Specifically, there is a scarcity of epidemic data with high-resolution spatial information, or the available data exhibit a granularity that is too coarse to effectively capture spatial nuances. Additionally, fitting epidemic data to network models or spatial diffusion equations proves to be highly challenging owing to the intricate nature of the model structures. Furthermore, the complexity of these model structures hinders the comprehensive analysis or prediction of fundamental epidemic indices, including peak values, final sizes and herd immunity.

For this reason, homogeneous compartment models are frequently used in scenario analysis to predict the epidemic trends of emerging infectious diseases due to the huge advantages of this modelling framework, given the aspects of parameterization, interpretability, ease of calibration, computational efficiency and strong predictive capabilities. Although spatial heterogeneity has been recognized in epidemic modelling for decades, most research has focused on modifying the transmission terms within homogeneous compartmental models to incorporate spatial heterogeneity. Consequently, various types of incidence rates have been proposed, revealing a range of complex dynamic behaviours [10,17,18]. However, the transmission terms describe a process in which there is no explicit spatial behaviour (no ‘movement parameters’), although spatial distribution and movement is implicitly incorporated. Subsequently, in various scenarios, the prediction accuracy of the existing homogeneous models may be compromised by oversimplification, as they tend to disregard important heterogeneity factors. Several recent studies [19,20] have developed explicit methods that incorporate spatial heterogeneity to quantify the spatio-temporal transmission of wildlife diseases. However, these methods require high-resolution spatial data. Therefore, exploring novel approaches that explicitly integrate spatial heterogeneity within homogeneous compartmental modelling frameworks to add new insights for modelling heterogeneity, particularly for precise (by involving heterogeneity) and efficient (by leveraging the advantages of homogeneous model framework) prediction of epidemics, is the scope of this study.

The main purpose of this study is to develop a generalized modelling framework that embeds spatio-temporal heterogeneity of the susceptible population into the homogeneous models. The ultimate aim is to leverage the homogeneous compartment model for conducting efficient prediction of epidemic trends, and simultaneously enhance the prediction accuracy of the epidemics. In the next section, we firstly develop a generalized framework, and design a machine-learning approach and pre-set a specific form to shape the recruitment process from the epidemic data of COVID-19. Subsequently, we apply our proposed model to project the epidemic trends of various COVID-19 variants as case studies, conducting tests and evaluations to compare prediction accuracy with traditional homogeneous modelling frameworks. Finally, we discuss the biological implications and the prospective applications of our novel modelling framework.

## Methods

### Model formulation

The classical *SIR* compartment model proposed by Kermack and McKendrick in 1927 [21,22] is:

$$\left\{\begin{array}{c} S\prime =-\beta_{0}cI\frac{S}{N},\\ I\prime =\beta_{0}cI\frac{S}{N}-\gamma I,\\ R\prime =\gamma I, \end{array}\right.$$ (1)

where *S*, *I*, *R* denote the susceptible, infectious, and recovered populations, respectively, *N* = *S* + *I* + *R* denotes the total population in the considered region, which is constant as *dN*/*dt* = 0. Here, we consider the variables as the absolute population while the units are listed in Table 1. The parameter *γ* is the recovery rate. The term $\beta_{0}c\frac{S}{N}I$ is the standard incidence rate for describing the transmission of the disease. That is, each infectious individual can generate a constant number of contacts *c* per unit time with a transmission probability *β*_0_ per contact, correspondingly, *β* = *β*_0_*c* is usually called the transmission rate. In addition, *S*/*N* is the probability at which each infectious individual contacts with susceptible individuals, because only the contacts between infectious and susceptible individuals can transmit the pathogen.

**Table 1:** Definitions and values of parameters and variables in model (3) and model (4).

	Definition	Values: mean (95% CI)		Units	Source
		UDE Model	Mechanistic model		
Parameters					
*β*	*β* = *β*_0_*c* (transmission rate)	0.4743	0.4945 (0.4943, 0.4946)	Day^-1^	Estimated
*β*_0_	Transmission probability per contact	--	--	--	--
*c*	Constant contact rate per day	--	--	Day^-1^	--
*γ*	Recovery rate	1/5		Day^-1^	Ref [35]
*k*	*k* = *ηp*	--	0.2812 (0.2810, 0.2814)	--	Estimated
*p*	Probability that the contact happens outside the epicentre	--	--	--	--
*η*	Proportionality factor	--	--	--	--
*R*_0_	Initial (basic) reproduction number	2.37	2.47	--	Estimated
Variables					
*N*(0)	Total population	2.475*10^7^		People	Database
*S*_*r*_(0)	Initial reserve susceptible population	*N*(0) − *S*_*e*_(0)		People	Calculated
*S*_e_(0)	Initial effective susceptible population	400002	409999 (409997, 410001)	People	Estimated
*I*(0)	Initial infections	1.778	0.7041 (0.7005, 0.7078)	People	Estimated
*R*(0)	Initial recovered populations	0	0	People	Assumed

The fundamental premise underlying homogeneous models is that the population is homogeneously mixing, so the proportion of infectious individuals around any susceptible individual is equal regardless of the location of the individual in the area. Consequently, when considering a single new outbreak of an emerging infectious disease, the entire population is considered as the initial condition for the susceptible class, i.e. (*S*(0) = *N*). Over the past few decades, the SIR model has undergone extensive extensions to various frameworks, such as SEIR and SEIHR models. However, most of these extensions have retained the assumptions related to managing the susceptible population [23–27]. As discussed in the introduction, such assumptions are deemed unreasonable due to their overlooking of spatial distances between individuals.

**Fig 2:**
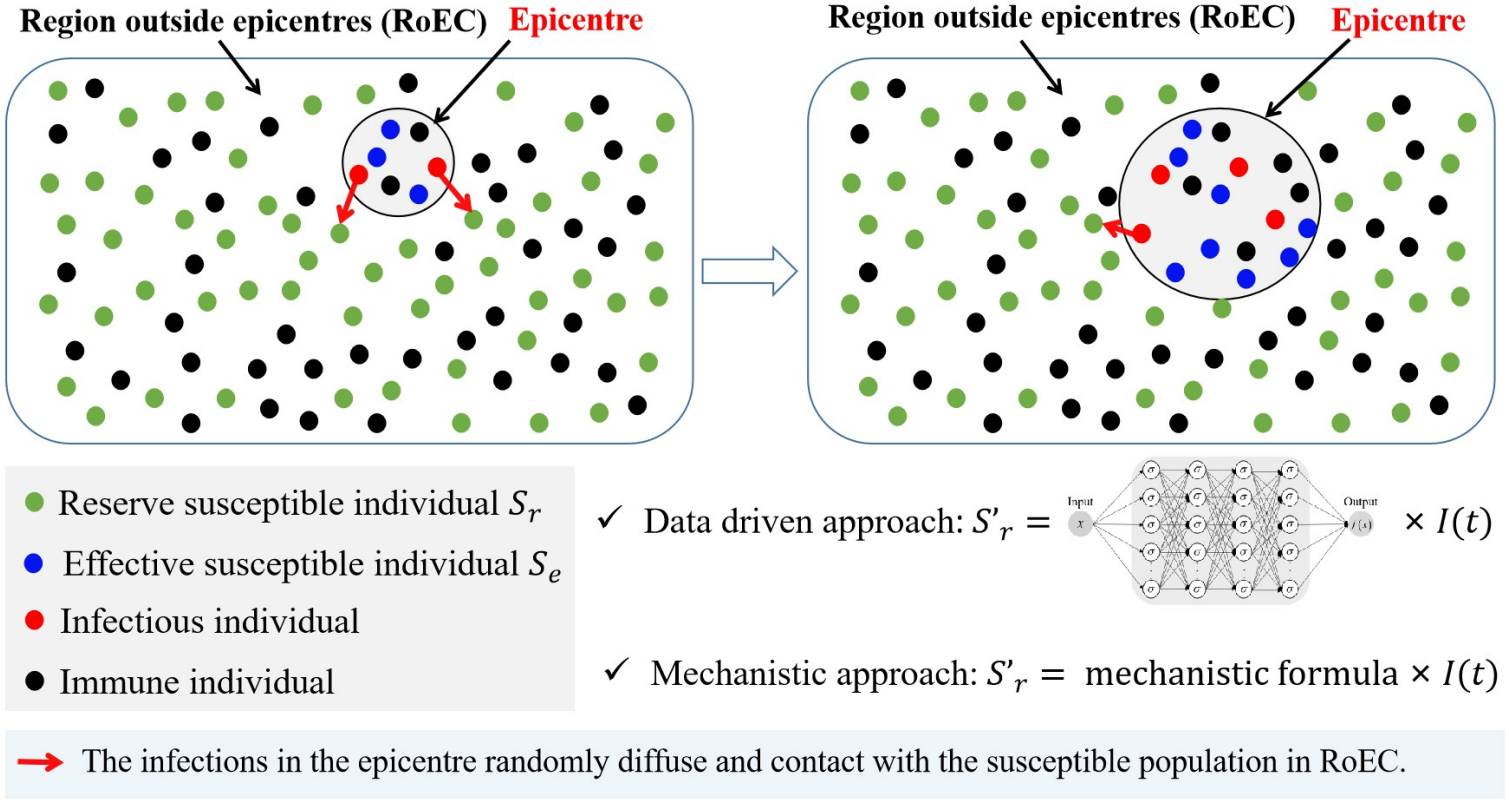
Schematic diagram illustrating the recruitment process of susceptible individuals. Within the epicentre, the classic SIR compartment model remains applicable for describing the transmission of the pathogen. The crucial aspect lies in incorporating the recruitment process of the susceptible population into the classic SIR compartment model, recognizing the spatio-temporal heterogeneity of the susceptibles. We term the susceptible individuals in the epicentre as the effective susceptible population, denoted by *S*_*e*_, and those in RoEC are referred to as the reserved susceptible population, denoted by *S*_*r*_. Considering the continuous spatial spread of infections, the epicentre is expanded to encompass more susceptibles. As a result, the susceptible population in class *S*_*r*_ will transition into the newly added epicentre and become *S*_*e*_. Letting *f*(*t*) represent the transition rate from *S*_*r*_(*t*) to *S*_*e*_(*t*), we establish the following generalized modelling framework in terms of the transition process of susceptibles.

To establish a more realistic modelling framework, we posit that transmission only occurs among the population within a small neighbourhood of all the infectious individuals, who are also assumed to be uniformly mixed within the neighbourhood. Therefore, the transmission among the population within the neighbourhood can be appropriately modelled within the homogeneous compartment model framework, specifically concerning the transmission of the pathogen through physical contacts. In this context, an area under consideration can be spatially divided into two distinct regions: the neighbourhood of all the infections, referred to as the epicentre, and the remaining region outside the epicentre (RoEC), as illustrated in Fig 2. As depicted in Fig 1, the evolution of diseases reveals the spatial diffusion of infected cases since the initiation of the outbreak. Therefore, the transmission should be initially considered within a small region that covers all the infectious individuals, i.e. the epicentre, and this will be expanded as the spatial diffusion of infectious individuals progresses. That is, attributed to the spatial diffusion of infectious individuals, the infections in the epicentre can randomly contact with the susceptible population in RoEC, the region of the epicentre should then be expanded and consequently more susecptibles will be involved in the transmission process, as shown in Fig 2. This is in line with the modelling idea of reaction-diffusion epidemic models with free boundary [28, 29].

$$\left\{\begin{array}{c} S_{r}^{\prime }=-f\left(t\right),\\ S_{e}^{\prime }=f\left(t\right)-\frac{\beta S_{e}I}{N_{e}},\\ I\prime =\frac{\beta S_{e}I}{N_{e}}-\gamma I,\\ R\prime =\gamma I. \end{array}\right.$$ (2)

where *N* = *S*_*r*_ + *S*_*e*_ + *I* + *R* denotes the total population in the considered region, which is a constant as *dN*/*dt* = 0. The population involved in the transmission process at time *t*, i.e. *N*_*e*_(*t*) = *S*_*e*_(*t*) + *I*(*t*) + *R*(*t*), is assumed to be uniformly mixed, hence we use the standard incidence rate $\frac{\beta S_{e}I}{N_{e}}$ to describe the transmission process of the pathogen between infectious individuals and susceptible individuals.

### Estimation of the transition rate of *f*(*t*)

Next, we focus on determining and estimating *f*(*t*). Susceptibles are involved in the transmission process because the infectious individuals make contact with the susceptible population outside the epicentres. Therefore, it is reasonable to assume that a larger population of infectious individuals should have a higher probability of contacting individuals in RoECs, consequently the transition rate at time *t* (i.e. *f*(*t*)) should be proportional to the current infections (i.e. *I*(*t*)), that is *f*(*t*) ∝ *I*(*t*).

Several advanced methods, such as Universal Differential Equations (UDEs) [30] and Physics-Informed Neural Networks (PINNs) [31], have been developed to integrate mechanistic models with neural networks. These well-established and extensively validated components are directly incorporated into the mechanistic model, while the neural network is employed to approximate complex and less-well-understood functions. *f*(*t*) should have a complex form but there is very limited information and existing experience on the form of the function. Therefore, the approach coupling the mechanistic model with neural networks is an attractive way to shape the transition rate from data directly. It should be noticed that PINN introduce physical laws into the training process as part of the loss function, while it uses neural networks to approximate and solve the variables (such as *S*(*t*), *I*(*t*), *R*(*t*) in this study). In contrast, UDEs, which originate from neural ordinary differential equations, maintain the compartmental frameworks but introduce a neural network solely to approximate unknown functions, thus staying much closer to a traditional compartmental modelling framework. For this reason, we initially employ the UDE framework and the technology of deep learning to shape the form of *f*(*t*) from epidemic data.

The approach of UDEs helps to avoid unreasonable assumptions by allowing the neural networks to learn functions directly from the data, however, it has a poor performance when attempting generalization to conduct scenario analysis under different situations due to the lack of a mechanistic form. Therefore, it is also essential to find a special form of the function, which should be also in line with the shape learned from UDEs. Both symbolic regression and cutting-edge neural networks (Kolmogorov-Arnold Networks (KANs)) [32] provide feasible methods to learn a mechanistic form, however, both of these methods have a poor robustness when reconstructing the functions, and such reconstructed functions are usually complex, hence not intuitive. Alternatively, we try to pre-set the specific form of *f*(*t*) and estimate the parameters in *f*(*t*) by fitting the model to the epidemic data and the output data of *f*(*t*) by the UDEs, simultaneously.

### Universal differential equation-based model

Here, we present a data-driven approach designed to utilize epidemic data for shaping the time-varying recruitment rate of susceptibles. As mentioned above, given the limited information on the transition rate, the UDE modelling framework can be a very good choice for learning the recruitment pattern of susceptibles from epidemic data. That is, utilizing the universal approximation property of deep neural networks, we use a neural network to present *f*(*t*) in the absence of the specific form of it, and then train the neural network to shape the specific form of *f*(*t*) for particular outbreaks of infectious diseases. Incorporating the assumption of *f*(*t*) ∝ *I*(*t*), we therefore have *f*(*t*) = *r*^*NN*^(*t*) * *I*(*t*) with *r*^*NN*^(*t*) being the neural network to approximate the time-varying recruitment rate of the susceptible population from the RoECs to the epicentres. Consequently, we propose the following universal differential equations model based on the generalized modelling framework (model (2)) [30,33]:

$$\left\{\begin{array}{c} S_{r}^{\prime }=-r^{NN}\left(t\right)*I,\\ S_{e}^{\prime }=r^{NN}\left(t\right)*I-\frac{\beta S_{e}I}{N_{e}},\\ I\prime =\frac{\beta S_{e}I}{N_{e}}-\gamma I,\\ R\prime =\gamma I. \end{array}\right.$$ (3)

By transferring the training process of the UDE model to solve an optimal control problem, we can extend the idea of back-propagation of neural networks to train the neural network included in the UDE model, in differential programming with the fully fledged technique of adjoint sensitivity analysis [34].

### Mechanistic model

We also try to find a particular form of the time-varying recruitment rate for understanding the transition mechanism of the susceptible population. As mentioned above, each infectious individual can generate a constant contact rate *c* per unit time. We then set *p* as the probability of the contacts happening outside their epicentre, hence, there are in total *pcI*(*t*) contacts outside the epicentre at time *t*. Similar to the standard incidence rate, contacts should also happen between infectious individuals *I*(*t*) and the reserved susceptible population *S*_*r*_(*t*) for successfully introducing new infections in RoEC and enlarge the epicentre, and the corresponding probability is given by *S*_*r*_/*N*. Then, it is reasonable to assume that the recruitment rate should be proportional to the newly acquired infections in the RoEC. Consequently, we have the following form of the recruitment rate:

$$f\left(t\right)=f\left(S_{r}\left(t\right),I\left(t\right)\right)=\eta *\beta_{0}*pcI*\frac{S_{r}}{N}=k\beta I\frac{S_{r}}{N},$$

where *k* = *ηp* with *η* being the proportionality factor and *β* = *β*_0_*c*. Obviously, the above form of the recruitment rate satisfies the basic assumption that *f*(*t*) is proportional to the current infections *I*(*t*) with $\frac{\partial \text{f}}{\partial \text{I}}>0$. Further, there is $\frac{\partial \text{f}}{\partial {\text{S}}_{\text{r}}}>0$, indicating that the recruitment rate is also increasing as there are more susceptibles in the RoEC. Then, the generalized model (2) has the following specific form

$$\left\{\begin{array}{c} S_{r}^{\prime }=-k\beta I\frac{S_{r}}{N},\\ S_{e}^{\prime }=k\beta I\frac{S_{r}}{N}-\frac{\beta S_{e}I}{N_{e}},\\ I\prime =\frac{\beta S_{e}I}{N_{e}}-\gamma I,\\ R\prime =\gamma I. \end{array}\right.$$ (4)

The definitions and values of all the parameters and variables for both the mechanistic model and the UDE model are given in Table 1.

### Data and data processing

**Fig 3:**
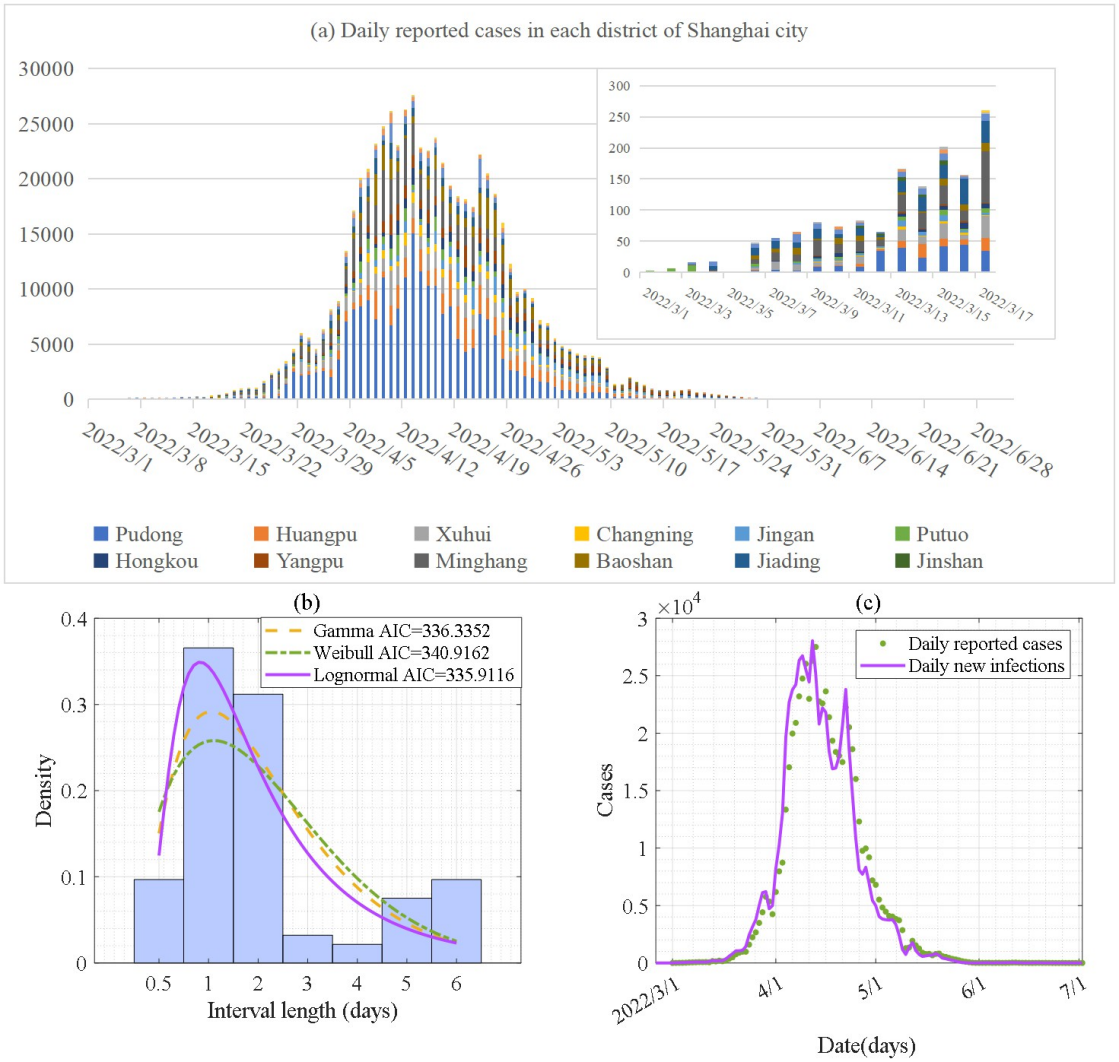
Data and processing data. (a) Time series of the numbers of daily reported cases of the Omicron variant in the 16 districts of Shanghai city from 1 March to 2 July 2022. (b) Frequency distribution histogram of the time-interval from infection to report and the fitting results of the probability distribution. (c) Processed data of the numbers of daily new infections.

We collected the epidemic data on the local outbreak of the Omicron variant of COVID-19 starting at Shanghai city, China, from 1 March to 2 July 2022 from the Shanghai Municipal Health Commission [36]. There are 16 districts in Shanghai city, namely, Pudong, Huangpu, Xuhui, Changning, Jingan, Putuo, Hongkou, Yangpu, Minhang, Baoshang, Jiading, Jinshan, Songjiang, Qingpu, Fengxian and Chongming districts, as shown in Fig 1. Correspondingly, we obtained the time series of the numbers of daily reported cases in each district, and the time series of the total daily numbers of reported cases in the whole of Shanghai city, as shown in Fig 3a. We also obtained part of the epidemiological survey data during 1 to 9 March 2022 from the Shanghai Municipal Health Commission [36], which includes 93 confirmed cases with the report date and the date that they closely contacted infections. Consequently, we can obtain the time-interval from the date of infection (physical contact) to the date of the report for the 93 confirmed cases, and the frequency distribution histogram of the time-interval is shown in Fig 3b.

We then processed the data to generate the time series of the numbers of daily new infections for model calibration as we did not consider the incubation period in the proposed model. We therefore firstly use three commonly used probability distributions, i.e. Gamma, Weibull, and Log-normal distributions, to fit the probability distributions of the time-interval from infection to report, as shown in Fig 3b. With the standard of minimizing the AIC, the Log-normal distribution was selected as the best choice. Based on the above distribution, we can further use the deconvolution method to obtain the numbers of daily new infections by processing the time series of the numbers of daily reported cases [37–39]. In detail, the number of daily new infections is denoted by $N_{j}^{I}$, where *j* = *t*_1_,…, *t*_*k*_ with *k* being the length of the time series. The $N_{j}^{I}$ are independent of each other and follow the Poisson distribution with mean *λ*_*j*_, i.e.$N_{j}^{I}\sim P(\lambda_{j})$. The probability of the numbers of infected cases on day *j* being reported on day *i* is denoted by *p*_*ij*_, which corresponds to the time interval from infection to report and was obtained by discretization of the Log-normal distribution as shown in Fig 3b. By incorporating the time series of the numbers of daily reported cases *C*_*i*_, *i* = *s*_1_,…, *s*_*m*_ (*m* is the length of the time series), we can obtain the point estimate value ${\hat{\lambda }}_{j}$ for $N_{j}^{I}$ by iterating the following formula:

$$C_{i}^{n}=\overset{i}{\underset{j=t_{1}}{\sum }}\;\lambda_{j}^{n}p_{ij},\lambda_{j}^{n+1}=\frac{\lambda_{j}^{n}}{q_{j}}\overset{S_{m}}{\underset{i=max\left\{j,S_{1}\right\}}{\sum }}\;\frac{p_{ij}C_{i}}{C_{i}^{n}},$$

where *n* is the number of iterations, $q_{j}=\sum_{i=\text{max}\left\{j,s_{1}\right\}}^{s_{m}}p_{ij}>0$, *j* ∈ [*t*_1_, *t*_*k*_]. The iterative procedure stops when the fitting error $\frac{1}{m}\sum_{i=s_{1}}^{s_{m}}\frac{{\left(C_{i}^{n}-C_{i}\right)}^{2}}{C_{i}^{n}}$ is small enough [37–39]. Through the above process, the processed data of the time series of the numbers of daily new infections are shown Fig 3c.

### Model training and calibration

We used the numbers of daily new infections in the local outbreak of the Omicron variant in Shanghai city to train the UDE model (i.e. model (3)) and we also calibrated the mechanistic model (model (4)). To this end, we firstly fixed the recovery rate as *γ* = 1/5 informed from the literature [35]. Before 1 March 2022, Shanghai city mainly experienced the wild type and Delta variants of COVID-19 with cumulative infected cases being less than 0.2% of the total population [36], hence the initial number in the recovery class is assumed to be 0 when the Omicron variant circulated for the first time in March 2022 in Shanghai, and the total population of Shanghai city was *N* = 2.475*10^7^. As a consequence, we only need to estimate the number of initial infections (*I*(0)), the initial effective susceptible population (*S*_*e*_(0)), the transmission rate (*β*) and the parameter *k* for the mechanistic model (model (4)), and the parameters in the neural network of the UDE model (model (3)).

To train the UDE model, the loss function is defined as:

$${\text{Loss}}_{\theta }=\sum_{\text{t}=1}^{\text{n}}{\left({\text{Data}}_{\text{t}}-\int_{\text{t}-1}^{\text{t}}\frac{{\beta \text{S}}_{\text{e}}\left(\text{t}\right)\text{I}\left(\text{t}\right)}{{\text{N}}_{\text{e}}\left(\text{t}\right)}\right)}^{2},$$

where $\int_{\text{t}-1}^{\text{t}}\frac{{\beta \text{S}}_{\text{e}}\left(\text{t}\right)\text{I}\left(\text{t}\right)}{{\text{N}}_{\text{e}}\left(\text{t}\right)}$ is the number of daily new infections at time t solved by the UDE model (model (3)), *θ* is the number of unknown parameters to be estimated, including the parameters in the neural network, the transmission rate *β*, and the initial conditions of *S*_*e*_(0), *I*(0). Then, training of model (3) is actually defined as solving the following optimal control problem:

$$\begin{array}{l} \underset{\theta }{min}\;Loss_{\theta }\Leftrightarrow \underset{\theta }{\text{min}}\sum_{\text{t}=1}^{\text{n}}{\left({\text{Data}}_{\text{t}}-\int_{\text{t}-1}^{\text{t}}\frac{{\beta \text{S}}_{\text{e}}\left(\text{t}\right)\text{I}\left(\text{t}\right)}{{\text{N}}_{\text{e}}\left(\text{t}\right)}\right)}^{2},\\ \text{s}.\text{t}.\;{\text{S}}_{\text{e}}\left(\text{t}\right),\;\text{I}\left(\text{t}\right),\;{\text{N}}_{\text{e}}\left(\text{t}\right)\;\text{satisfy}\;\text{model}\;\left(3\right) \end{array}$$

Deep learning processes for training the UDE model were implemented in the open source Julia language 1.9.2. In particular, we defined a two-layer neural network, where the first layer has an input length of 1 (i.e. time *t*) and an output length of 32 and the second layer has an input length of 32 and an output length of 1 (i.e. the recruitment rate at time *t* (*f*(*t*))). We used Tanh as the activation function, and the fifth order explicit Runge-Kutta method to solve the neural network differential equations. In training the model, we set the batch size = 1 and optimized 500 times using the Adam optimizer with an initial learning rate = 0.02 due to the different lengths of the input temporal data. Then the model was optimized 2000 times using BFGS.

When fitting the data to model (4), the objective function *F* is defined as the residual sum of squares between the real data of the time series of the number of daily new infections, the recruitment rate estimated by the UDE model to the correspondingly predicted numbers by solving model (4), hence:

$$F=\sum_{t=1}^{n}{\left(Data_{t}-\int_{t-1}^{t}\frac{\beta S_{e}\left(t\right)I\left(t\right)}{N_{e}\left(t\right)}\right)}^{2}+\sum_{t=1}^{n}{\left(f_{UDE}\left(t\right)-k\beta I\left(t\right)\frac{S_{r}\left(t\right)}{N\left(t\right)}\right)}^{2},$$

where $\int_{t-1}^{t}\frac{\beta S_{e}\left(t\right)I\left(t\right)}{N_{e}\left(t\right)}$ is the number of daily new infections projected by the mechanistic model (model (4)) on day *t* while *Data*_*t*_ is the observed number of the data; *f*_*UDE*_(*t*) is the time series of the estimated recruitment rate by the UDE model; *n* denotes the sample size of the time series of the number of daily new infections. We used the adaptive Metropolis-Hastings (M–H) algorithm to carry out the Markov Chain Monte Carlo (MCMC) procedure for data fitting and parameter estimation of the mechanistic model [40]. The algorithm was run for 50,000 iterations with a burn-in of 300,000 iterations, and the Geweke convergence diagnostic method was employed to assess convergence of chains.

## Results

**Fig 4:**
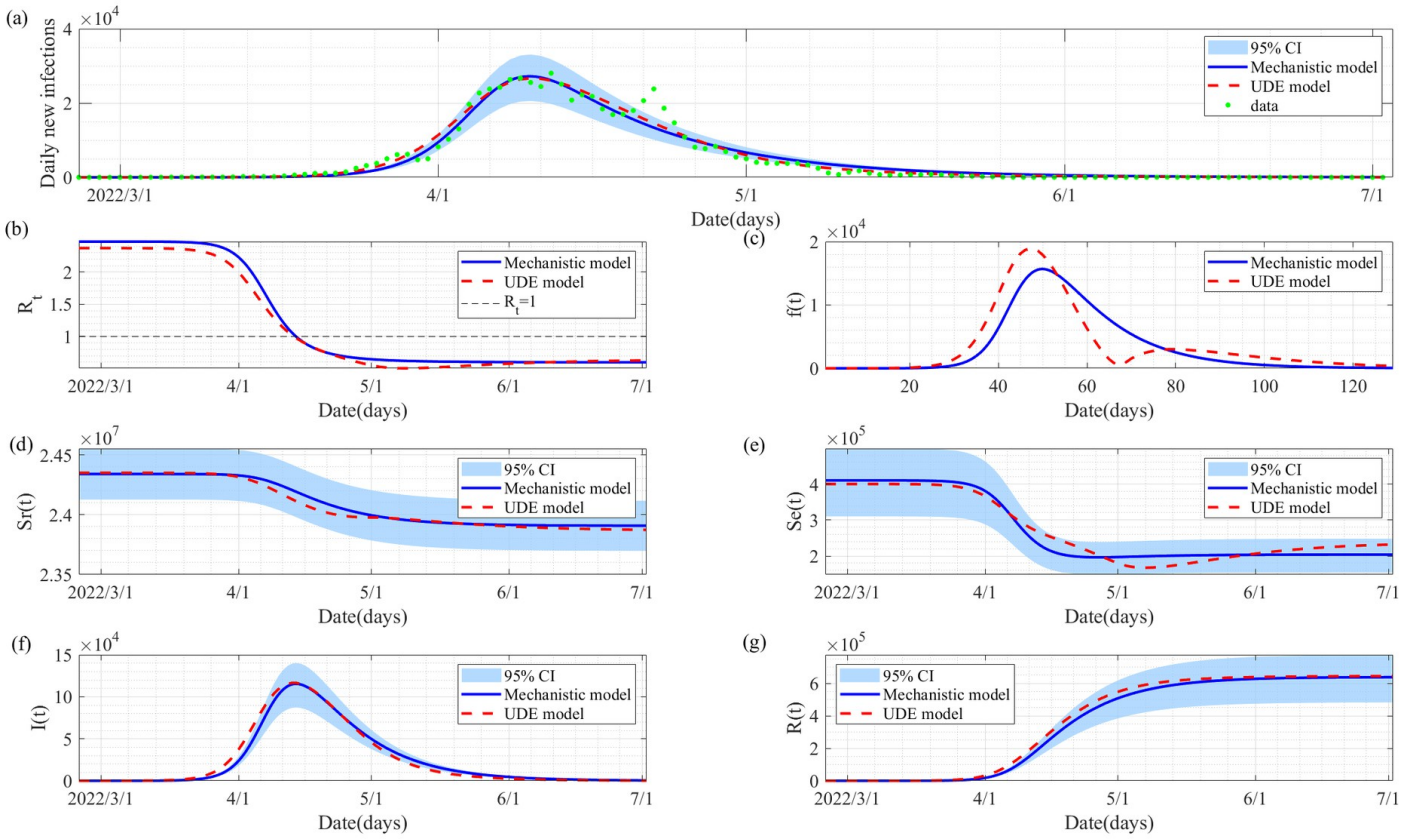
Model calibrations and solutions. **(a)** Training result of the UDE model (red dashed curve) and the best fitting result of the mechanistic model (blue solid curve), here the blue shaded area is the 95% confidence interval of the fitting result of the mechanistic model. **(b)** Estimated effective reproduction number from the two models. **(c)** Estimated recruitment rate of the susceptible population *f*(*t*). **(d)-(g)** Solutions of the UDE model and the mechanistic model by fixing the parameters and initial conditions as the estimated values listed in Table 1.

The training results of the UDE model and the best fitting results of the mechanistic model are shown in Fig 4a. Correspondingly the estimated values of the parameters and initial conditions are detailed in Table 1. It follows from Fig 4a that both the mechanistic model and the UDE model can fit the epidemic data of the numbers of daily new infections very well. The fitted curves of these distinct models consistently maintain proximity throughout the observed period. Of particular significance, Fig 4c shows the functions of the recruitment rate over time in both models. It is evident that the two curves exhibit a high degree of concordance, indicating a robust fit. This alignment supports the validity of our chosen formula in the mechanistic model for characterizing the enigmatic recruitment process of the susceptible population, aligning closely with patterns discerned from the epidemic data by the machine learning methods. Similar insights emerge upon scrutiny of the solutions for the reserved susceptible population and the effective susceptible population, as depicted in Fig 4d and 4e, respectively, for both the mechanistic and UDE models.

Notably, the size of the estimated initial effective susceptible population of the mechanistic model is approximately 409,999 As the COVID-19 spread in Shanghai city, the total numbers in the susceptible population involved in the epidemic wave escalated to 842,212. This implies that only a fraction of the Shanghai city population, specifically 3.4%, were in the population of actual susceptible individuals during the outbreak, which is characterized by an accumulation of 625,437 infections in reality. Therefore, this aspect offers a practical method for estimating the size of the actual susceptible population for a specific infectious disease outbreak, where the actual susceptible population equals the entire susceptibles minus the final reserved susceptibles *S*_*r*∞_.

**Fig 5:**
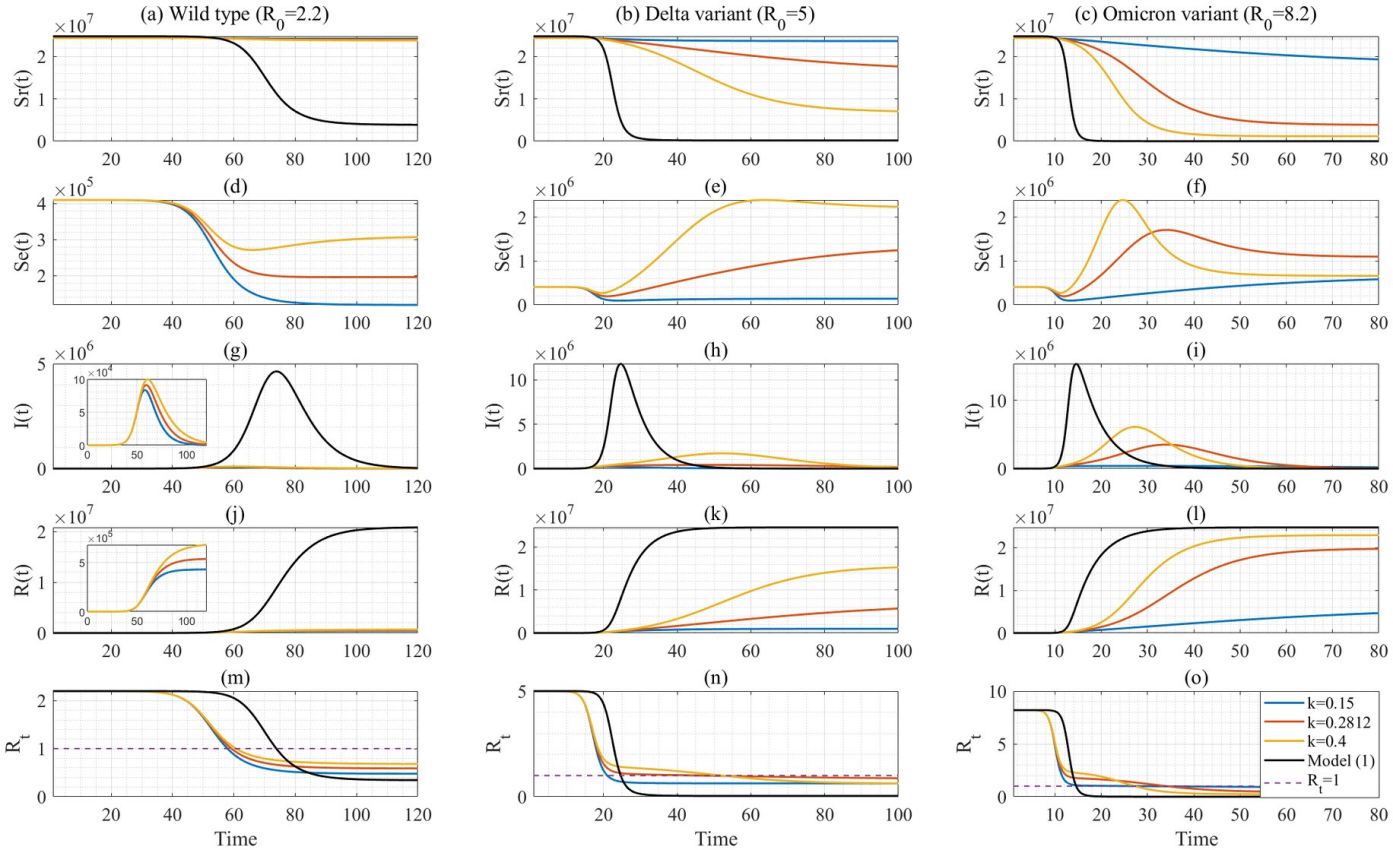
Solutions of the mechanistic model (model (4)) with three different values of *k* and the homogeneous model (model (1)) by varying the transmission rate *β* (corresponding to different variants of COVID-19). Here, we plotted the solution of *S*(*t*) of model (1) in the first panel. The corresponding effective reproduction numbers are shown in the last panel.

Subsequently, we conduct scenario analysis, in terms of *k* and the transmissibility *β* associated with distinct variants of COVID-19, to show in depth how the heterogeneity affects the transmission dynamics, by comparing the difference between the epidemic curves produced by the novel mechanistic model (model (4)) and the homogeneous model (i.e. model (1)) by choosing the same parameter values for the two models. By fixing the recovery rate *γ* = 1/5 for both model (1) and model (4), and choosing the basic reproduction number as 2.2 [41], 5 [42], and 8.2 [35,43] for the wild type, Delta and Omicron variants of COVID-19, the transmission rate *β* can be calculated as 0.44, 1 and 1.64, respectively, for the two models. The initial conditions were fixed as the same as those of the estimation results outlined in Table 1. Consequently, we present the solutions of model (4) for different variants with varying basic reproduction numbers in Fig 5. Additionally, Table 2 compiles the corresponding peak size, attack rate, final size, and herd immunity for each scenario.

**Table 2:** Prediction of key epidemic indices for different scenarios using model (4).

	Indices	*k* = 0.15	*k* = 0.2812	*k* = 0.4	Model (1)
		Unit: million			
*R*_0_ = 2.2	Final size (Attack rate)	0.4295 (1.74%)	0.5393 (2.18%)	0.6928 (2.80%)	20.8775 (84.35%)
	Peak of daily new infections (Rate to population)	0.0194 (0.08%)	0.0208 (0.08%)	0.0222 (0.09%)	1.0971 (4.43%)
	Peak of infection class (Rate to the population)	0.0836 (0.34%)	0.0911 (0.37%)	0.0996 (0.40%)	4.6346 (18.73%)
	Final reserve susceptible population *S*_*r*∞_	24.2011	24.0141	23.7476	3.8725
	Herd immunity	1.02%	1.17%	1.34%	54.38%
*R*_0_ = 5	Final size (Attack rate)	0.9814 (3.97%)	6.9588 (28.12%)	15.6843 (63.37%)	24.5790 (99.31%)
	Peak of daily new infections (Rate to the population)	0.0815 (0.33%)	0.0914 (0.37%)	0.3602 (1.46%)	4.4472 (17.97%)
	Peak of infection class (Rate to the population)	0.2413 (0.98%)	0.4148 (1.68%)	1.7257 (6.97%)	11.8363 (47.82%)
	Final reserve susceptible population *S*_*r*∞_	23.6269	16.3921	6.8531	0.1710
	Herd immunity	1.82%	10.70%	35.67%	79.42%
*R*_0_ = 8.2	Final size (Attack rate)	6.0057 (24.27%)	19.8149 (80.06%)	22.9198 (92.61%)	24.7432 (99.97%)
	Peak of daily new infections (Rate to the population)	0.1516 (0.61%)	0.7838 (3.17%)	1.5282 (6.17%)	8.1122 (32.78%)
	Peak of infection class (Rate to the population)	0.3962 (1.60%)	3.5199 (14.22%)	6.1219 (24.73%)	15.3837 (62.16%)
	Final reserve susceptible population *S*_*r*∞_	18.0593	3.8424	1.1676	0.0068
	Herd immunity	7.30%	49.61%	63.67%	86.97%

Note: Attack rate = Final size/Total population. Herd immunity is defined here as the ratio of the total number of immune individuals (cumulative infections) to the population at the date when the effective reproduction number decreases to 1.

Based on the findings from Table 2 and Fig 5, when the basic reproduction number is *R*_0_ = 8.2 for the Omicron variant, we observe that a substantial ratio of the susceptible population (representing 84.48% of the total population) actively participates in the transmission process during the large-scale epidemic wave for *k* = 0.2812. In contrast to the homogeneous model, which assumes the entire population as being the initial number of susceptibles, the proposed model, incorporating the recruitment process of susceptible populations, significantly reduces the projected cumulative number of infections for the large-scale epidemic wave by 19.91% (i.e., 99.97% for the homogeneous model versus 80.06% for our model) for *R*_0_ = 8.2.

Furthermore, if we take the point at which the effective reproduction number reaches unity as the time when the population attains herd immunity leading to the decline in new infections, we find that cumulative infections at that point amount to 49.61% of the population (i.e. the herd immunity) in our proposed model when *R*_0_ = 8.2 and *k* = 0.2812. In comparison, the homogeneous model has already reached 86.97% of the population by that date when *R*_0_ = 8.2. Similarly, it can be seen from Table 2 and Fig 5 that the peak sizes of daily new infections and the infectious class experience a substantial reduction, i.e. reduced by 29.61% and 47.94% for *R*_0_ = 8.2, respectively, when spatio-temporal heterogeneity is incorporated.

**Fig 6:**
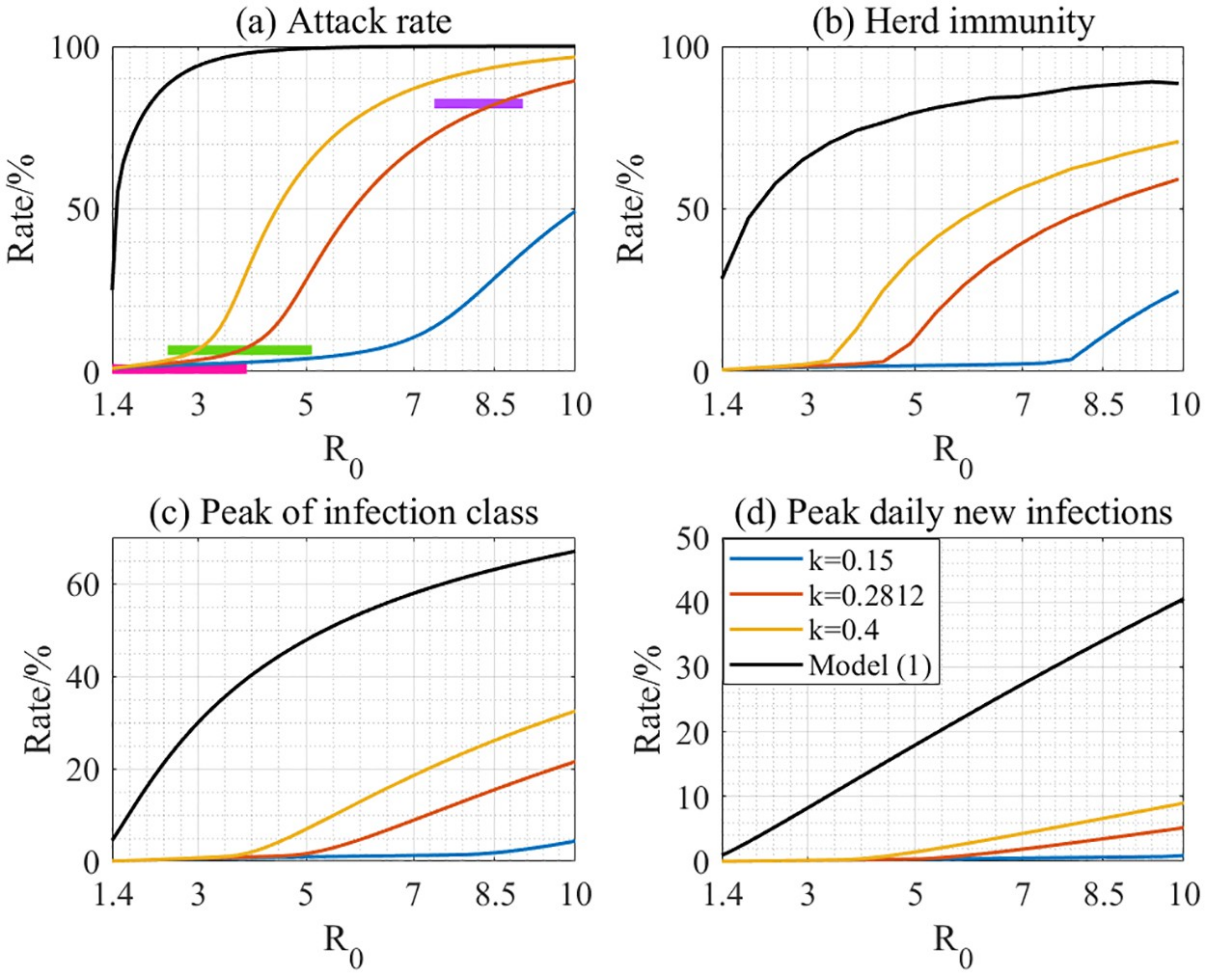
Comparison of four epidemic indices predicted by models (4) and (1), with the basic reproduction number varied between 1.4 and 10. The horizontal bars represent the reference values of the attack rate for the wild type with *R*_0_ ∈ (1.4, 3.9) [41], Delta variant with *R*_0_ ∈ (2.43, 5.11) [42], and Omicron variant strains with *R*_0_ ∈ (7.4, 9) [43], respectively. The reference value for Omicron is derived from survey data in [44]. The values for the wild type strain and the Delta variant were calculated by analyzing the rates of epidemics in Belarus under weak intervention measures. The data from 1 March to 1 October 2020, are used for the wild type strain, and data from 1 October 2020, to 20 December 2021, are used for the Delta variant.

In conclusion, with spatio-temporal heterogeneity considered, the peak size, final size and herd immunity corresponding to outbreaks of emerging infectious diseases are all significantly diminished as shown in Fig 6. The potential reason is that a significant proportion of susceptibles is not initially involved in the transmission process during the early phase, and when they are gradually recruited into the transmission process, the former infected and recovered population, as a public immunity barrier, can offer cross-protection, thereby mitigating the risk of infection for the newly involved susceptibles. That is, the temporal heterogeneity of the susceptibles joining the transmission process contributes to the decrease of peak size, final size and herd immunity.

Additionally, upon examining the values presented in Table 2 across various variants of COVID-19, it becomes evident that the disparities in both the projected peak size and the final size between models with and without spatio-temporal heterogeneity become more pronounced as the basic reproduction number decreases from 8.2 to 2.2. This phenomenon can be more intuitively seen from Fig 6a. To illustrate this, when we fix *k* = 0.2812, the difference in attack rate projected by the two models with and without heterogeneity are 0.82, 0.71, 0.20 for *R*_0_ = 2.2, 5, 8.2, respectively. In essence, this implies that for viruses (or other pathogens) with low transmissibility, the heterogeneity of the susceptible population can exert a significantly more discernible impact on the transmission dynamics of infectious diseases. This may also be attributed to the fact that as the reproduction number decreases, a considerably smaller proportion of the entire population become actual susceptibles during a single epidemic wave.

### Prediction accuracy assessment

On 7 December 2022, China’s National Health Commission announced major changes to the country’s policies on COVID-19, marking a notable shift away from its zero-COVID policy. As a result, China experienced a significant COVID-19 epidemic wave [45,46] starting from that point. On 25 January 2023, the China CDC released a report detailing the national situation on novel coronavirus infections. The report highlighted that the positive rate of infections detected by nucleic acid tests peaked on 22 December 2022, and by 23 January 2023, it had decreased to a very low level, signalling the end of the first epidemic wave after the easing of the zero-COVID policy. After the large scale outbreak, a recent study conducted four online surveys across 31 provincial-level administrative divisions in China to gather data on infection status, indicating that as of 7 February 2023, approximately 82.4% of the Chinese population had been infected [44]. Similarly, a large scale cohort study, by enrolling 14,744 participants in Shaanxi province of China, revealed a cumulative infection attack rate (IAR) of 84.7% for COVID-19 among the study participants after the easing of the zero-COVID policy [47]. Taking the survey data on the cumulative infection rate (attack rate) as the reference, the prediction accuracy of our model is defined as follows:

$$\text{Prediction}\;\text{Accuracy}=100\text{\%}-\frac{\left|CI_{data}-CI_{model}\right|}{CI_{data}}*100\%,$$

where *CI*_*data*_ is the real value from data and *CI*_*model*_ is the projected value from the model.

It follows from Table 2 that without considering the heterogeneity of the susceptibles, the attack rate projected by model (1) is 99.97% for the large-scale outbreak of the Omicron variant with a basic reproduction number of 8.2. Theoretical studies also indicate that the final epidemic size in the traditional *SIR* model exceeded 99% of the population when the basic reproduction number reached 8.2 [48]. However, this outcome is markedly inconsistent with the actual situation reported in the aforementioned studies [44,47]. In contrast, our modelling framework, by involving the spatio-temporal heterogeneity, estimates the attack rate to be around 80.06% when *R*_0_ = 8.2. Taking 82.4% as the reference, the prediction accuracy of our model reaches 97.2% while the prediction accuracy of the homogeneous model is 78.6%, i.e. resulting in an 18.6% improvement in the prediction accuracy.

Further, when the basic reproduction number is 8.2, the homogeneous model projects the peak size of daily new infections as 32.78%, which would be a huge number given the large scale outbreak of the Omicron variant in China and is out of line with the real case. Instead, our model projects a peak of around 3.17% of the population, aligning more closely with the estimated 4.7% of the population for the large-scale outbreak of the Omicron variant in Beijing city after the easing of the zero-COVID policy [45]. Moreover, an intuitive observation from Fig 5i and 5l reveals that the outbreak duration is approximately 70 days when we fix k = 0.2812 as the estimated value, whereas the homogeneous model projects a shorter duration of 30–40 days. Notably, from the announcement of major changes of control policy on 7 December 2022 to 23 January 2023 (the end of the epidemic wave), the first large-scale epidemic wave in China after the easing of the dynamic zero-COVID policies actually persisted for 77 days, which is also consistent with our projection.

In Fig 6, we further show the differences in the predictions of model (1) and model (4) by varying the basic reproduction number from 1.4 to 10. The horizontal bars represent the attack rate calculated from data, which results from the possible range of the basic reproduction number. Therefore, it can be intuitively seen that the prediction of the attack rate for model (4) is much closer to the reference data marked as a purple bar in Fig 6a, indicating a significant improvement in prediction accuracy. Furthermore, we collected data on COVID-19 epidemics in the Republic of Belarus from Our World in Data (https://ourworldindata.org/coronavirus), where weak control interventions were implemented. The attack rate for the epidemics of the wild type strain and the Delta variant were around 0.8% and 6.6%, respectively, marked as rose red and green bars in Fig 6a. Using these data as a reference, we can intuitively see from Fig 6a the prediction accuracy for both the wild type strain and the Delta variant is also significantly improved in our model by incorporating heterogeneity.

In summary, by incorporating spatio-temporal heterogeneity into the classical homogeneous model, our model, in an efficient way, accurately depicts the epidemic curve of the first large-scale outbreak of the Omicron variant in China, including peak size, final size, herd immunity, and outbreak duration. Furthermore, the prediction accuracy is significantly enhanced compared to the homogeneous model.

## Discussion

The presence of population heterogeneity is ubiquitous and represents a key factor in modelling infectious diseases, exerting a significant influence on transmission dynamics and epidemic outcomes [49–52]. Therefore, the development of effective modelling frameworks that account for spatial heterogeneity remains a central focus. Traditionally, patch models, dynamic network models, and diffusion models have been commonly employed to characterize the spatial spread of infectious diseases over time. However, their utility in conducting quantitative analyses of epidemic outbreaks, grounded in real-world data, is impeded by the inherent complexity and high dimensionality of these model structures in terms of the mechanistic modelling framework. This challenge has motivated us to devise a modelling framework characterized by a simplified structure, specifically designed to accommodate spatio-temporal heterogeneity. The objective is to render feasible the projection of key epidemic indices and enhance the overall performance of mathematical models under the influence of spatio-temporal heterogeneity, facilitating a more robust understanding of infectious disease dynamics.

As a solution, we introduced a pioneering modelling framework that incorporates spatio-temporal heterogeneity into traditional compartment models, thereby leveraging the inherent advantages of the compartmental modelling framework. This integration facilitates the precise quantification and prediction of critical epidemic parameters such as peak size, herd immunity, and final size of each epidemic wave. Our approach involved shaping the spread process of the susceptibles during a specific outbreak of the Omicron variant of COVID-19 in Shanghai, China, using two distinct methodologies. Employing the cutting-edge technology of deep learning, we trained our Universal Differential Equation (UDE) model and utilized the Markov Chain Monte Carlo (MCMC) method for calibrating the underlying mechanisms. The deep learning method played a crucial role in validating the assumptions embedded in the specific forms of the mechanistic model. Concurrently, the mechanistic model contributed to uncovering potential mechanisms in the recruitment process of susceptibles. Additionally, it addressed the limitations of the data-driven approach based on deep learning, particularly in terms of enhancing the generalization performance for conducting scenario analyses and predictions. This dual-method strategy synergistically combines the strengths of deep learning and mechanistic modelling to yield a comprehensive and robust framework for understanding and precisely predicting infectious disease dynamics.

A comparison of the estimated results for the transition rate shows that the deep learning-based data-driven approach is more flexible in capturing detailed information. Notably, there is a slight increase around day 65 in Fig 4c. The potential reason for this is that the reported numbers of infected cases suddenly rose during the decreasing phase of the epidemic wave (see Fig 4 for details), resulting in the increase of the recruitment rate of the susceptible population. This phenomenon also suggests the possibility that the diffusion of infections may lead to multiple instances of herd immunity, as the increased recruitment rate can cause the effective reproduction number to rise and exceed the threshold again. Even so, the mechanistic model can offer intuitive insights into the transmission and control of infectious diseases. The parameter *k* can be interpreted as the spatial diffusion speed in some contexts. In addition, we know that numerous factors, such as human behavioural changes, contact structures, and the evolution of the pathogen, influence the spread of infectious diseases. For simplicity, the impact of many of these factors on disease transmission can be represented by the transmission rate in epidemic models. For example, behavioural changes for self-protection can be reflected as a reduction in the transmission rate. Notably, the transmission rate parameter is incorporated into our mechanistic term for the recruitment rate of susceptibles. This offers an effective method for understanding and quantifying how various factors influence the recruitment process of susceptibles during an outbreak. Additionally, it helps to explain how control interventions affect the spatial spread of infections, providing an alternative approach to controlling infectious diseases. That is, a higher diffusion speed and larger transmission rate result in an increased recruitment rate of susceptibles, consequently leading to a higher attack rate, a peak in daily new infections, and a larger herd immunity, as can be intuitively seen in Fig 6. Therefore, interventions aimed at reducing the transmission capacity and spatial spread of the disease should be critical in controlling infectious diseases.

Through the incorporation of heterogeneity into the homogeneous model, we have observed a significant enhancement in prediction accuracy, with an approximately 18.6% improvement when assessing the cumulative attack rate during the large-scale outbreak of the Omicron variant in China, after the relaxation of the dynamic zero-COVID policy. Notably, when considering peak size, herd immunity, and the outbreak period, our proposed model exhibits projections that also closely align with real-world scenarios. Note that the outcomes of our study underscore that peak size, final size, and herd immunity in real-world scenarios are notably smaller than predictions derived from homogeneous models. Epidemic models are crucial tools that support decision-making in controlling infectious diseases. For instance, they help to determine the necessary preparation of medical resources during the peak of each epidemic wave or the vaccination coverage needed to achieve herd immunity. Therefore, accurate predictions of epidemic curves are essential for effective and cost-efficient outbreak management of infectious diseases. Consequently, the inclusion of heterogeneity becomes crucial for accurately estimating the requirements for medical resource preparation during large-scale outbreaks of emerging infectious diseases, as well as determining the necessary vaccination coverage. Our modelling framework presents a practical and effective method for precisely addressing these considerations. This highlights the importance of incorporating our modelling method into various compartmental modelling frameworks to enhance the prediction accuracy of epidemic models, ultimately improving decision-making in the control of infectious diseases.

Our novel framework of the mechanistic model (i.e. model (4)) adopts a remarkably simple method to incorporate heterogeneity into mathematical models. Consequently, this simplicity facilitates the extension of the modelling framework to consider various aspects of infectious disease transmission with spatio-temporal heterogeneity in the susceptible population. Moreover, this modelling structure opens the door to conducting more theoretical studies aimed at qualitatively analyzing the impact of heterogeneity on the transmission dynamics of infectious diseases and exploring optimal control strategies. In the light of these considerations, we contend that this study can serve as a foundation for the development and theoretical analysis of a class of models within the realm of modelling the heterogeneity of susceptible populations. The straightforward approach taken in our framework not only enhances its practical utility but also encourages further exploration and refinement, thereby contributing to the advancement of understanding and modelling infectious disease dynamics with spatio-temporal heterogeneity.

There are still several limitations to this study. Firstly, we only used one time-series of the epidemic data to train or calibrate the models, hence we appeal for the collection of more spatial data for calibrating our models, which would definitely help to increase the robustness of the results. Secondly, there is also another kind of heterogeneity (such as the age-structure of the population) which is ignored in the current study and how the different forms of heterogeneity reciprocally affect the transmission dynamics of infectious disease would be a very interesting issue. Finally, when we tried to generate the time series of the numbers of daily new infections, the frequency distribution of the time-interval from infection to report seems, potentially, to be bimodal, while our pre-set distributions were not bimodal. How the distribution of this time-interval will affect the results would also be an interesting issue. Furthermore, we did not consider demographic dynamics in our current model, as our focus is on a single outbreak of an emerging infectious disease. However, extending our modelling framework to incorporate the impact of demographic changes on the recruitment process of susceptibles, and subsequently on transmission dynamics, presents a very interesting topic for future research.
